# Identification of key gene networks controlling organic acid and sugar metabolism during watermelon fruit development by integrating metabolic phenotypes and gene expression profiles

**DOI:** 10.1038/s41438-020-00416-8

**Published:** 2020-12-01

**Authors:** Muhammad Jawad Umer, Luqman Bin Safdar, Haileslassie Gebremeskel, Shengjie Zhao, Pingli Yuan, Hongju Zhu, M. O. Kaseb, Muhammad Anees, Xuqiang Lu, Nan He, Chengsheng Gong, Wenge Liu

**Affiliations:** 1 Zhengzhou Fruit Research Institute, Chinese Academy of Agricultural Sciences, Henan Joint International Research Laboratory of South Asian Fruits and Cucurbits, Zhengzhou, China; 2 Key Laboratory of Biology and Genetics Improvement of Oil Crops, Oil Crops Research Institute, Chinese Academy of Agricultural Sciences, Ministry of Agriculture and Rural Affairs, Wuhan, 430062 China

**Keywords:** Secondary metabolism, Gene expression

## Abstract

The organoleptic qualities of watermelon fruit are defined by the sugar and organic acid contents, which undergo considerable variations during development and maturation. The molecular mechanisms underlying these variations remain unclear. In this study, we used transcriptome profiles to investigate the coexpression patterns of gene networks associated with sugar and organic acid metabolism. We identified 3 gene networks/modules containing 2443 genes highly correlated with sugars and organic acids. Within these modules, based on intramodular significance and Reverse Transcription Quantitative polymerase chain reaction (RT-qPCR), we identified 7 genes involved in the metabolism of sugars and organic acids. Among these genes, *Cla97C01G000640*, *Cla97C05G087120* and *Cla97C01G018840* (*r*^*2*^ = 0.83 with glucose content) were identified as sugar transporters (*SWEET*, *EDR6* and *STP*) and *Cla97C03G064990* (*r*^*2*^ *=* 0.92 with sucrose content) was identified as a sucrose synthase from information available for other crops. Similarly, *Cla97C07G128420*, *Cla97C03G068240* and *Cla97C01G008870*, having strong correlations with malic (*r*^*2*^ = 0.75) and citric acid (*r*^*2*^ = 0.85), were annotated as malate and citrate transporters (*ALMT7*, *CS*, and *ICDH*). The expression profiles of these 7 genes in diverse watermelon genotypes revealed consistent patterns of expression variation in various types of watermelon. These findings add significantly to our existing knowledge of sugar and organic acid metabolism in watermelon.

## Introduction

Watermelon is the fifth most consumed fleshy fruit worldwide, with a global yield of 118 million tons in 2017^1^. It occupies 7% of the total cultivated land of fruits and vegetables. Fruits of watermelon are rich in water and nutrients (amino acid sugars, carotenoids, lycopene, organic acids, etc.)^2,3^. These phytochemicals make watermelon one of the most nutritious fruits, providing substantial nutritional supplementation to the human diet^4–7^. Sugars and organic acids have a strong influence on organoleptic fruit quality and are crucial components involved in the development of fruit flavor^8^. Unlike staple food crops, for which yield is the ultimate breeding goal, taste and aroma are more vital traits for watermelon, which are both determined by the metabolite composition of the fruit. During the development process, watermelon fruits undergo numerous biochemical changes, including changes in sugar metabolism, organic acid and pigment accumulation, fruit softening, flavor, and aromatic volatile contents^9^.

Organic acids are the key players in maintaining pH and changes in the sensorial quality of fruit^10^. Assessment of fruit maturity depends on the sugar to acid ratio along with the quality of a certain cultivar^11^. The major sugars and organic acids in watermelon are fructose and malic acid^12^. Transport of sucrose to the fruit occurs via the phloem as part of the sugar metabolic pathway, and then neutral invertase converts sucrose into glucose, and fructose or sucrose synthase converts sucrose into UDP-glucose and fructose^13,14^. Two enzymes, namely, hexokinase and fructokinase, are involved in the phosphorylation of fructose and glucose into fructose 6 phosphate (F6P) and glucose 6 phosphate (G6P), respectively. Phosphoglucoisomerase is the enzyme responsible for catalyzing the interconversion of F6P and G6P in a reversible reaction. Special transporters confined to the vacuolar membrane can facilitate the transport of sucrose to the vacuole, while acid invertase can convert it into fructose and glucose^13^. These changes are mainly controlled by genetic factors, including variations in individual gene expression patterns and metabolic gene networks^15,16^. Changes that genetically regulate variations among the organic acids in fruits during their development have been reported for many fruits, including peach, lemon, pineapple, apple, and strawberry.

Advances in omics approaches along with quantitative biology offer several ways to identify gene networks and their regulatory mechanisms in living systems, and one such promising approach for identifying coexpressed gene networks using mRNA-Seq data is the weighted gene coexpression network analysis (WGCNA)^17^. WGCNA is useful for identifying modules/networks of coexpressed genes, correlating these modules with phenotypic traits and detecting key genes within the networks. This method has been previously applied to discover the coexpressed genes responsible for fruit acidity^18^, taste, and aromatic qualities of apricot^19^ and flavanol biosynthesis compounds in apple^20^. However, no such comprehensive studies are available to explain the gene networks and molecular regulatory mechanisms underlying organic acid and sugar regulation in watermelon fruit. Here, we used RNA sequencing data to uncover the regulatory networks and mechanisms controlling sugars and organic acids in sour watermelon (SrW) and sweet watermelon (SwtW) at 10, 18, 26, and 34 days after pollination. The differentially expressed genes (DEGs) and coexpression network analysis revealed gene networks linked to the metabolism of acids and sugars.

## Materials & methods

### Plant material

For this experiment, we selected two kinds of watermelon materials, namely, sweet watermelon (203Z) and a sour watermelon (SrW), to evaluate the variations in sugar and organic acid metabolic content. The details of the plant materials used in this study were explained in our previous report^21^. Briefly, the pure inbred line watermelon cultivar “203Z” accumulates high total soluble sugar contents and low total organic acid contents at maturity. “SrW”, developed by crossing the inbred cultivar “203Z” and the wild subspecies “PI271769”, accumulates low total soluble sugar contents and high total organic acid contents. The plant materials were cultivated at the Horticultural Research Base in Henan, China, in spring 2018. Plants were sown following a randomized complete block design with 10 plants in each row and a 50 cm distance between rows. Self-pollination was performed manually for the female flowers, and to record the date of pollination, watermelon plants were tagged. Moreover, samples from watermelon flesh were collected at 4 different developmental stages (10, 18, 26, and 34 DAP), immediately transferred to liquid nitrogen, and finally stored at −80 °C for further sugar and organic acid determinations and extraction of RNA for validation of RNA-Seq data.

### Measurement of total soluble solids and pH in watermelon flesh

After homogenizing collected watermelon flesh samples, TSS (Brix %) and pH were measured. For TSS measurements, a refractometer was used (HC-112ATC, Shanghai LICHENKEYI, China). pH was recorded by using a pH meter (PHB-4, Shanghai LICHENKEYI, China) at 10, 18, 26, and 34 days after pollination^22^.

### Quantification of organic acids and soluble sugars in watermelon fruit flesh

Watermelon samples were cut diagonally into two parts. Flesh samples were collected from both portions by carefully avoiding seeds in the middle portion. After taking flesh samples from both portions, the two parts were pooled together. Pooling was performed for the flesh material of two parts of the same sample. The samples were instantly frozen and then used to determine the organic acid (citric acid, oxalic acid, and malic acid) and soluble sugar (sucrose, fructose, and glucose) contents as described in previous reports^22,23^. Flesh samples were taken at 4 developmental stages (10, 18, 26, and 34 DAP) from 3 biological replicates.

### Construction and sequencing of the RNA-sequencing library

The RNA-Seq library was prepared by a previously described method^24^, and a 1% agarose gel was used to check for contamination and degradation of RNA. The purity of RNA was analyzed by using a Nano Photometer^®^ spectrophotometer (IMPLEN, CA, USA). Estimation of RNA concentration was performed using a Qubit^®^ RNA Assay Kit and a Qubit^®^ 2.0 Fluorometer (Life Technologies, CA, USA). The integrity of RNA was checked by using an Agilent Nano 6000 assay kit (Santa Clara, California, United States).

### Mapping to watermelon genome V2 and quantification of gene expression

Clean reads were aligned to the watermelon reference genome (http://cucurbitgenomics.org/organism/21) by using TopHat v2.0.12. Read counting in features (genes, in this case) was performed using HTSeq v0.6.1^25^. Gene lengths and read counts that were mapped to genes were used to calculate FPKM values^26^.

### Analysis of differential expression

The differentially expressed genes (DEGs) between SrW and SwtW were identified with the DESeq R package^27^, and Benjamini–Hochberg-adjusted *P*-values < 0.05 were considered statistically significant^28,29^.

### Enrichment analysis of DEGs and WGCNA for identifying correlated gene networks

KOBAS software was used to test for enrichment of DEGs in KEGG pathways^30^. WGCNA was performed in R using default parameters to simplify genes into coexpressed modules^17,31^. FPKM values were normalized, and an adjacency matrix was constructed. The phenotype data were imported into the WGCNA package, and correlation-based associations between phenotypes and gene modules were calculated using the default settings. The WGCNA package was used to convert the adjacency matrix into a topological overlap matrix (TOM). After constructing a network, the transcripts with identical patterns of expression were grouped into one module, and eigengenes were also calculated for these modules. The genes from each module were exported using the default parameters for Cytoscape export.

### Validation of intramodular candidates through RT-qPCR analysis

Gene expression analysis using RT-qPCR was carried out using three independent biological replicates for each sample^32^. Extraction of total RNA was performed by using a TIANGEN kit (Beijing, China). cDNA was synthesized by using a Takara Tokyo, Japan) kit with reverse transcriptase. The target candidate genes were selected from the gene modules based on their intramodular gene significance and the annotation information from the watermelon reference genome database (http://cucurbitgenomics.org/organism/21). Primer design and RT-qPCR were carried out as described previously^12^. Actin (*Cla016178*) was used as a reference gene^33^.

## Results

### Variations among soluble sugars during watermelon fruit flesh development

**Fig. 1:**
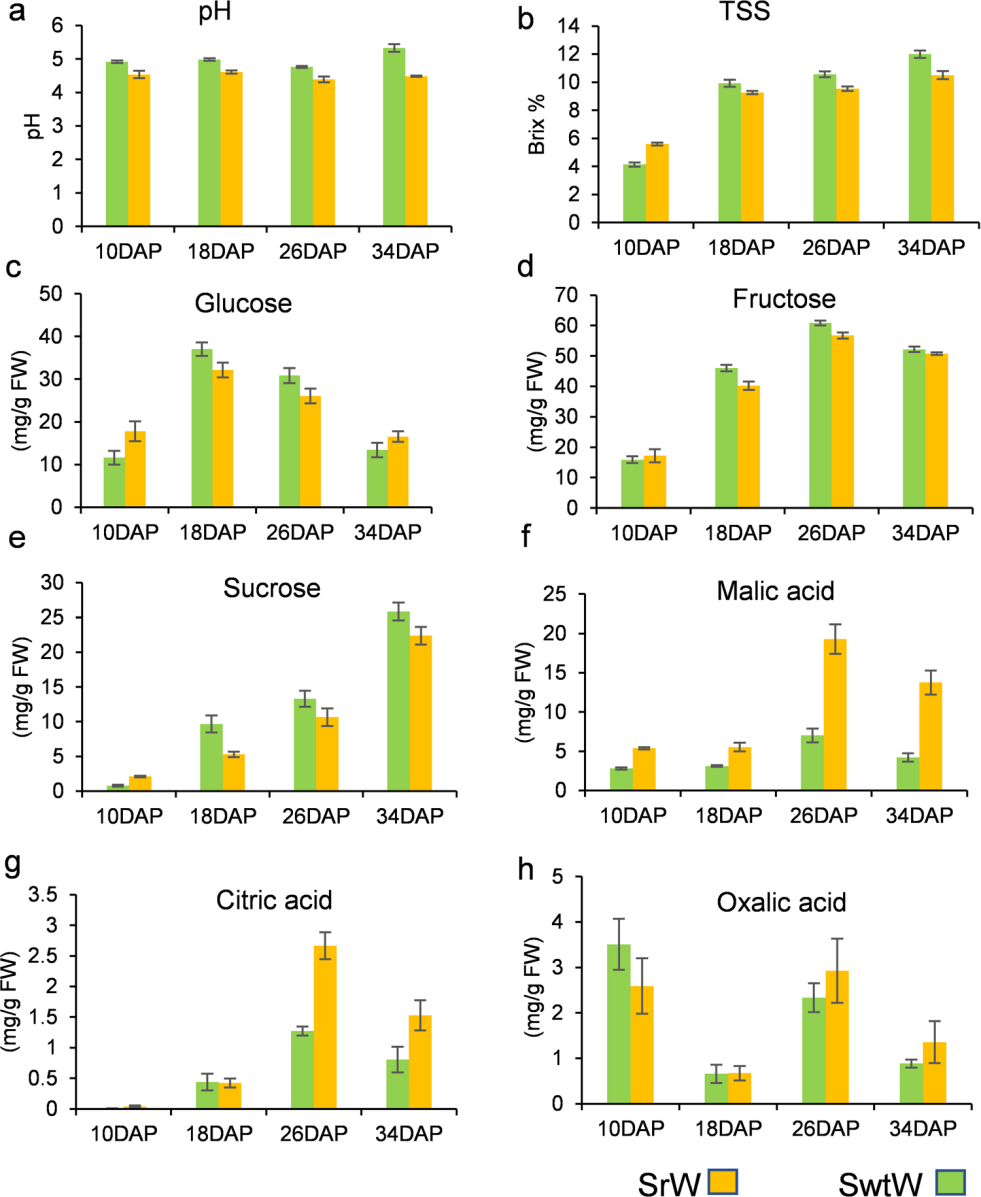
Total soluble solids (Brix %), pH, soluble sugars (glucose, sucrose, and fructose), and organic acids (malic, oxalic, and citric acid) (mg/g FW) in watermelon fruit at 10, 18, 26, and 34 DAP. Each value is the mean of three biological replicates

TSS was measured at 10, 18, 26, and 34 DAP. Significant variation was observed at different developmental stages. TSS increased from 4.13 and 5.60 Brix% at 10 DAP to 12 and 10.5 Brix% at 34 DAP in SwtW and SrW, respectively (Fig. 1a).

A significant difference was observed in soluble sugar content, i.e., glucose, sucrose, and fructose, over the course of watermelon fruit development. The glucose content was measured as 11.6, 37.01, 30.83, and 13.43 mg/g FW at 10, 18, 26, and 34 DAP in SwtW, while in SrW, it was 17.76, 32.15, 26.05, and 16.54 mg/g FW at 10, 18, 26, and 34 DAP, respectively. An increase in the glucose content was observed from 10 DAP to 18 DAP (37.01 and 32.15 mg/g FW), but at 34 DAP, the glucose content was the same as that observed at 10 DAP (13.42 mg/g FW in SwtW and 16.54 mg/g FW in SrW) (Fig. 1c). The same trend was observed for fructose at all stages of watermelon fruit development, i.e., 15.86, 46.04, 60.86, and 52.17 and 17.16, 40.17, 56.75, and 50.77 mg/g FW at 10, 18, 26, and 34 DAP in SwtW and SrW, respectively (Fig. 1d). These results confirmed that as the fruit moves towards maturity, fructose and glucose are broken down to generate the energy necessary for various metabolic processes and fruit ripening^11^. The sucrose content was very low at 10 DAP but increased significantly at 18 DAP and 26 DAP (9.67, 13.28, and 5.2, 10.60 mg/g FW) and 34 DAP (25.83 and 22.39 mg/g FW) in SwtW and SrW (Fig. 1e).

### Variations among organic acids throughout the development of watermelon fruit flesh

The difference in pH was recorded at different developmental stages. For example, SwtW had a pH of 4.92, 4.98, 4.76, and 5.33 at 10, 18, 26, and 34 DAP, respectively, whereas, in SrW, a pH of 4.53, 4.61, 4.38, and 4.48 was observed, respectively, at the 4 stages (Fig. 1b).

In the present study, malic acid was observed as the major acid in both materials. An increase in the malic acid content was observed from 10 DAP to 26 DAP in SwtW and SrW (Fig. 1f). At maturity, the malic acid content decreased but was still higher in SrW than in SwtW. The concentration of citric acid was extremely high at 26 DAP (Fig. 1g) but decreased at maturity. In the case of oxalic acid, the concentration was higher at 10 DAP in SwtW and SrW but decreased near maturity, i.e., to 0.88 mg/g FW in SwtW and 1.35 mg/g FW in SrW (Fig. 1h). Transcriptome analysis and identification of DEGs in watermelon fruit

For transcriptome profiling, samples were collected at 4 stages of watermelon fruit development (10, 18, 26, and 34 DAP) from 3 replicates of both SwtW and SrW to construct 24 cDNA libraries. In total, 191.86 Gb of clean data was filtered, and 6 Gb of clean data for each individual sample was obtained with more than a 91% Q30 base percentage. When sequencing data were aligned to the reference genome, more than 90.1% of clean reads were mapped. Less than 3% of the reads were not mapped (Table 1).

**Table 1:** RNA sequencing data and corresponding quality control information

Sample	Raw Reads	Clean Reads	Clean Bases (G)	Error Rate (%)	Q20 (%)	Q30 (%)	GC Content (%)
SrW-10-DAP-1	48182826	47528796	7.13	0.02	97.31	92.46	44.44
SrW-10-DAP-2	55137270	54423292	8.16	0.02	97.27	92.43	44.55
SrW-10-DAP-3	51336756	50749952	7.61	0.02	96.86	91.51	44.47
SrW-18-DAP-1	43412584	42284100	6.34	0.02	97.3	92.48	44.54
SrW-18-DAP-2	55231986	54005996	8.1	0.02	96.92	91.66	44.45
SrW-18-DAP-3	49853826	48583956	7.29	0.02	97.34	92.62	44.37
SrW-26-DAP-1	51969624	50987032	7.65	0.02	97.32	92.56	44.81
SrW-26-DAP-2	61879656	60922818	9.14	0.02	97.14	92.14	44.42
SrW-26-DAP-3	52478428	51496238	7.72	0.02	97.2	92.22	44.48
SrW-34-DAP-1	55758804	54620744	8.19	0.02	97.19	92.24	44.22
SrW-34-DAP-2	58035794	57123994	8.57	0.02	97.33	92.52	44.25
SrW-34-DAP-3	53535056	52656194	7.9	0.02	97.27	92.44	44.12
SwtW-10-DAP-1	54604124	53720332	8.06	0.02	97.29	92.52	44.5
SwtW-10-DAP-2	49493794	48856014	7.33	0.02	97.08	92.03	44.37
SwtW-10-DAP-3	55005720	54233580	8.14	0.02	97.21	92.29	44.6
SwtW-18-DAP-1	61711222	60749998	9.11	0.02	97.37	92.65	44.34
SwtW-18-DAP-2	45632140	44865534	6.73	0.02	97.41	92.71	44.39
SwtW-18-DAP-3	61597858	59575252	8.94	0.02	97.28	92.45	44.38
SwtW-26-DAP-1	61892510	60571612	9.09	0.02	97.17	92.21	44.15
SwtW-26-DAP-2	59389180	58397668	8.76	0.02	97.08	92.03	44.26
SwtW-26-DAP-3	48156512	47044710	7.06	0.02	97.2	92.3	44.24
SwtW-34-DAP-1	53999022	53054908	7.96	0.02	97.33	92.52	44.1
SwtW-34-DAP-2	56646000	55534476	8.33	0.02	97.11	92.1	44.15
SwtW-34-DAP-3	58827286	56987568	8.55	0.02	97.17	92.19	44.08

We compared the transcriptome profiles of SwtW and SrW at 4 fruit developmental stages to identify the DEGs during watermelon fruit development (Supplementary Fig. 1). The DEGs were filtered according to an expression level | log2 (fold-change) | > 1 and an adjusted *P*-value < 0.05 in each pairwise comparison. The downregulated DEGs were more abundant than the upregulated DEGs at each developmental stage, but the upregulated DEGs were more abundant than the downregulated DEGs when the developmental stages were compared between the two materials (Table 2).

**Table 2:** Up- and downregulated DEGs at 10, 18, 26, and 34 DAP in SrW and SwtW

Groups	Total DEGs	Downregulated	Upregulated
SrW-10-DAP_vs_SrW-18-DAP	3530	2456	1074
SrW-10-DAP_vs_SrW-26-DAP	6441	4389	2052
SrW-10-DAP_vs_SrW-34-DAP	7173	4846	2327
SrW-18-DAP_vs_SrW-26-DAP	3961	2535	1426
SrW-18-DAP_vs_SrW-34-DAP	4891	3208	1683
SrW-26-DAP_vs_SrW-34-DAP	1877	1162	715
SwtW-10-DAP_vs_SrW-10-DAP	274	66	208
SwtW-10-DAP_vs_SwtW-18-DAP	3571	2476	1095
SwtW-10-DAP_vs_SwtW-26-DAP	6438	4140	2298
SwtW-10-DAP_vs_SwtW-34-DAP	8426	5450	2976
SwtW-18-DAP_vs_SrW-18-DAP	377	66	311
SwtW-18-DAP_vs_SwtW-26-DAP	3762	2256	1506
SwtW-18-DAP_vs_SwtW-34-DAP	6620	4213	2407
SwtW-26-DAP_vs_SrW-26-DAP	639	306	333
SwtW-26-DAP_vs_SwtW-34-DAP	3379	2408	971
SwtW-34-DAP_vs_SrW-34-DAP	2153	570	1583

KEGG analysis of DEGs identified from pairwise comparisons between developmental stages during watermelon fruit development provided additional information about the enriched biological pathways, including secondary metabolite biosynthesis, starch and sucrose regulatory metabolism, pentose and glucoronate interconversions, ubiquitin-mediated proteolysis, terpenoid backbone biosynthesis, glycolipid metabolism, flavonoid biosynthesis, fatty acid biosynthesis, elongation and degradation, and ascorbate and aldarate metabolism (Supplementary Fig. 2). Based on the statistical significance criterion for multiple testing correction (adjusted *P*-value), phenylpropanoid biosynthesis, glycerophospholipid metabolism, MAPK-signaling pathway, secondary metabolite biosynthesis, metabolism of sucrose and starch, metabolic pathways, and plant-pathogen interaction pathways were significantly enriched (*q*-value ≤ 0.001) and displayed a constant increase in expression throughout watermelon fruit development (Supplementary Fig. 3).

### Identification of coexpressed gene networks and key candidates

**Fig. 2:**
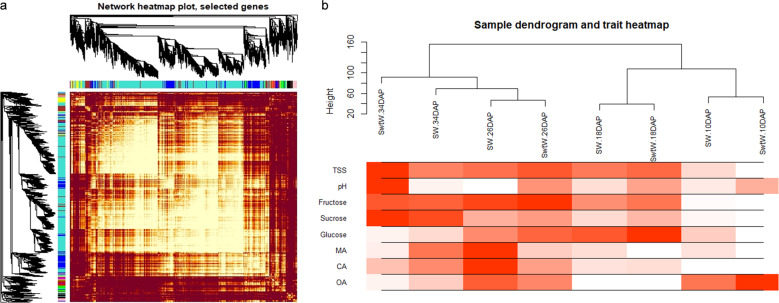
**a** Cluster dendrogram and network heatmap of genes subjected to coexpression module calculation. **b** Sample dendrogram and module trait heatmap at each developmental stage

From the transcriptome data entered into the WGCNA module with FPKM values, 11 distinct gene modules were identified based on the coexpression patterns of individual genes. These gene modules are labeled in distinct colors and presented as a clustergram and network heatmap (Fig. 2a). The TSS, pH, and glucose, sucrose, fructose, malic, citric, and oxalic acid contents at each developmental stage were used as phenotypic data for the analysis of module-trait correlations. A sample dendrogram and trait heatmap were constructed to illustrate the expression of each phenotypic parameter at different developmental stages (Fig. 2b).

**Fig. 3:**
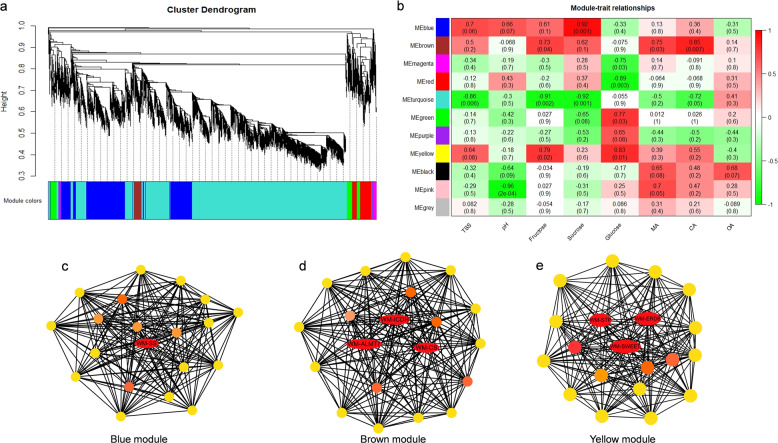
Gene networks and key candidate genes involved in sugar and organic acid regulation during watermelon fruit development as identified by WGCNA. **a** Hierarchical clustering presenting eleven modules having coexpressed genes. Each leaflet in the tree corresponds to an individual gene. **b** Module-trait associations based on Pearson correlations. The color key from green to red represents *r*^2^ values from -1 to 1. **c** Gene network for the blue module, which positively correlated with the sucrose content (*r*^2^ = 0.92, *P* = 0.001). **d** Gene network for the brown module, which positively correlated with the fructose (*r*^2^ = 0.73, *P* = 0.04), malic acid (*r*^2^ = 0.75, *P* = 0.03) and citric acid (*r*^2^ = 0.85, *P* = 0.007) contents. **e** Gene network from the yellow module, which positively correlated with the fructose (*r*^2^ = 0.79, *P* = 0.02) and glucose (*r*^2^ = 0.83, *P* = 0.01) contents. Hub genes (key candidates) within each network are highlighted in red due to the highest weight within the module and coded for gene descriptors based on annotations

Of the 11 coexpressed gene networks, 3 showed significant correlations with sugars and organic acids. The blue module contained 1301 genes and showed a significant association with sucrose with a correlation coefficient (*r*^2^) of 0.92. The yellow module with 410 genes was positively correlated with fructose (*r*^2^ = 0.79) and glucose (*r*^2^ = 0.83). The brown module consisting of 741 genes showed significantly high correlations with malic acid (*r*^2^ = 0.75) and citric acid (*r*^2^ = 0.85). A detailed description of the gene module and trait correlations is presented in Fig. 3a, b. The genes from each of these 3 modules were selected, and their edges and nodes were calculated with the WGCNA R package for gene-network visualizations.

To further search for candidate genes with major contributions within the gene networks, annotation information of all these genes was extracted from the watermelon reference genome annotation database. This integrative approach of combining intramodular hub genes consistent with DEGs and comparisons with annotation information was followed by RT-qPCR validation of 23 selected genes for precise identification of key candidates. These genes comprised 15 genes involved in the metabolism of sugar, 6 genes linked to malic acid metabolism, and 2 genes linked to citrate metabolism. The results obtained from the RT-qPCR and RNA-Seq data were consistent with each other (Supplementary Fig. 4). Finally, 3 genes (*Cla97C01G000640*, *Cla97C05G087120* and *Cla97C01G018840)* from the yellow module, correlated with glucose (*r*^2^ = 0.83), were identified as sugar transporters (*SWEET*, *EDR6* and *STP*), and 1 gene (*Cla97C03G064990*) correlated with sucrose (*r*^*2*^ *=* 0.92) was identified as a sucrose synthase (*SS*) from the information available for other crops. Similarly, 3 genes (*Cla97C07G128420*, *Cla97C03G068240*, and *Cla97C01G008870*) from the brown module, having a strong correlation with malate (*r*^2^ = 0.75) and citrate (*r*^2^ = 0.85), were annotated for malate and citrate transporters (*ALMT7*, *CS*, and *ICDH*, respectively). These putative candidates are highlighted in red in the gene networks (Fig. 3c–e). The genes described as key candidates were selected based on (1) intramodular significance (hub genes from modules correlated with sugars and organic acids) and (2) the gene expression profiles. In addition to the 7 genes predicted as key candidates, other hub genes from coexpression modules are listed with their annotations in Table 3, Supplementary Fig. 5, and Supplementary File 1.

**Table 3:** Hub genes selected from all the coexpression modules

ID	Module	Weight	Annotation
Cla97C02G029240	Green	0.44361	Chlorophyll a-b binding protein, chloroplastic
Cla97C01G009340	Green	0.40516	Transmembrane protein 56-like
Cla97C03G061330	Magenta	0.28378	Unknown protein
Cla97C03G061320	Magenta	0.1703	WRKY transcription factor 10
Cla97C07G143330	Pink	0.24742	Protein RIK
Cla97C01G005090	Purple	0.11057	Mitochondrial import inner membrane translocase subunit
Cla97C08G159290	Purple	0.13573	Alanine racemase
Cla97C06G121220	Purple	0.21865	Kinase family protein
Cla97C09G176620	Purple	0.17084	Forkhead-associated (FHA) domain
Cla97C07G130860	Purple	0.10818	Zinc finger CCCH domain-containing protein 2-like
Cla97C08G152340	Purple	0.10057	Kinase family protein
Cla97C03G058250	Purple	0.24516	Transmembrane protein, putative
Cla97C05G094950	Purple	0.13404	Lipase
Cla97C11G213440	Purple	0.1664	UDP-glycosyltransferase
CLa97C10G191460	Purple	0.19075	Receptor-like kinase
Cla97C04G072780	Purple	0.15719	Protein kinase-like protein
Cla97C05G105620	Red	0.12895	Ribosome maturation factor rimP
Cla97C02G046860	Turquoise	0.12046	NEDD8-specific protease 1
Cla97C01G024650	Black	0.13402	Protein TIFY 5A
Cla97C03G064990	Blue	0.44119	Sucrose synthase
Cla97C03G068240	Brown	0.21008	ATP-citrate synthase alpha chain protein 2
Cla97C07G128420	Brown	0.31835	Aluminum-activated malate transporter
Cla97C01G008870	Brown	0.2061	Isocitrate dehydrogenase [NADP]
Cla97C05G087120	Yellow	0.15897	sugar transporter ERD6-like 16
Cla97C01G018840	Yellow	0.21624	Sugar transporter, putative (DUF1195)
Cla97C01G000640	Yellow	0.19395	Bidirectional sugar transporter SWEET

### Genes related to soluble sugar metabolism

**Fig. 4:**
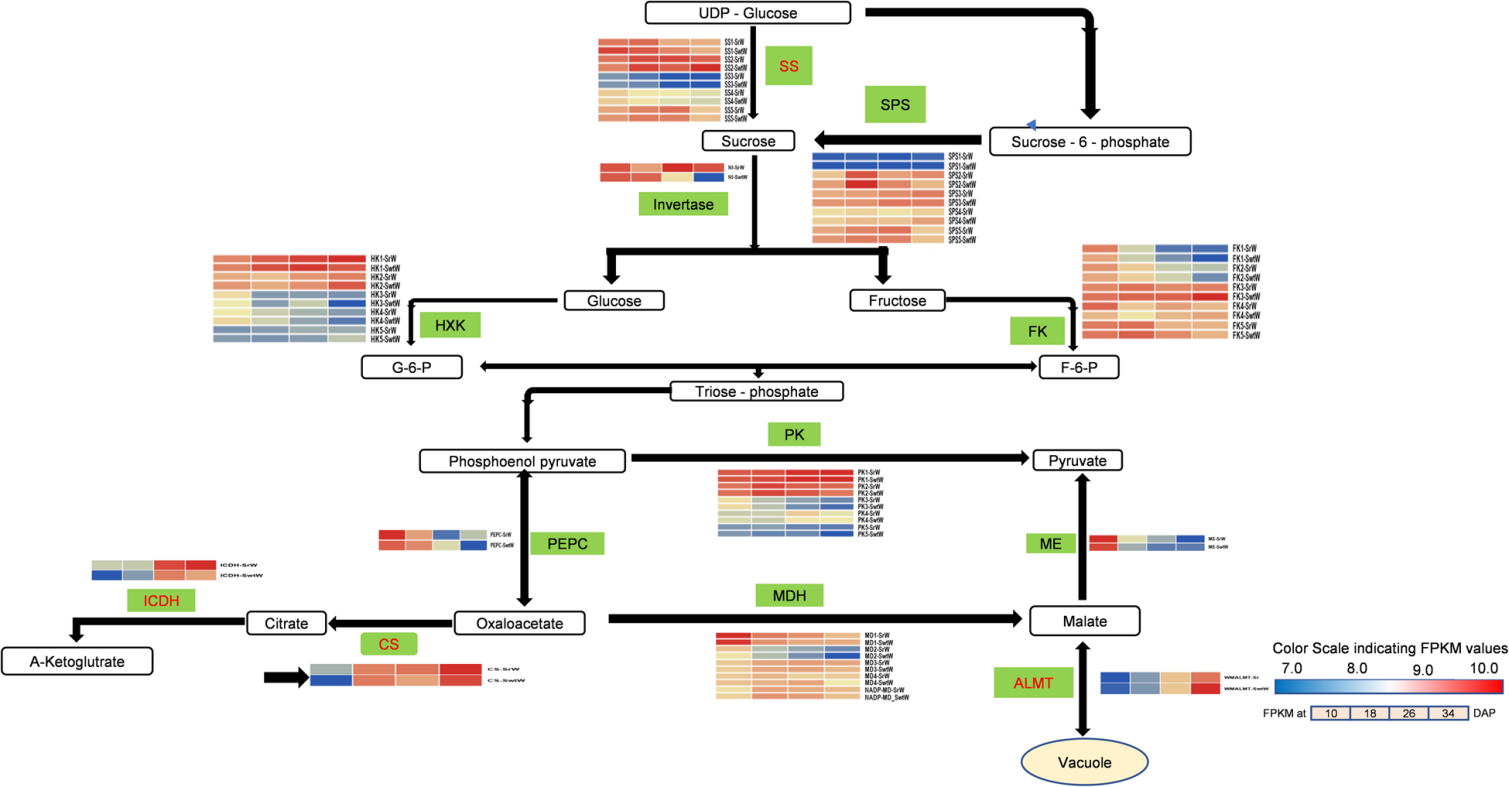
The organic acid and sugar biosynthetic pathway. Enzyme names are shown along with their expression patterns at various stages. Grids represent the expression patterns of genes shown as FPKM values: 10, 18, 26, and 34 DAP, left to right. The absolute expression is represented by the grids at 10, 18, 26, and 34 DAP, with FPKM values of 0–1, 1–2, 2–4, 4–8, 8–16, 16–32, 32–64, 64–128, 128–256, 256–512, 512–1200 and >1200

Some of the important genes that were identified as associated with sugar metabolism had a considerable impact on the traits measured at various stages of fruit development. Five genes linked to SS and 4 genes associated with SPS genes showed differential expression between SrW and SwtW. One of these sucrose synthase genes *(Cla97C03G064990)* was also present in the blue module that strongly correlated (*r*^2^ = 0.92) with sucrose content. The expression patterns of 2 sucrose phosphate synthase genes *(Cla97C06G120770* and *Cla97C11G215270)* were consistent with that of the sucrose content at 4 developmental stages. Five fructokinase (FK), 4 fructose bisphosphate aldolase (FBA), and 4 fructose 1-6, bisphosphate (FBP) genes were found to be differentially expressed at various stages of watermelon fruit development. The expression patterns of *FK (Cla97C05G094630)*, *FBA (Cla97C04G076620)* and *FBP (Cla97C08G147060)* were consistent with the fructose content at different developmental stages. Neutral invertase *(Cla97C05G088310)* was downregulated. Among the 5-hexose kinase (HK) genes, *Cla97C03G063270*, and *Cla97C11G208480* were upregulated. Of the 12 *SWEET* genes screened, only *WMSWEET1 (Cla97C01G000640)* was highly upregulated throughout the development of watermelon fruit. Expression of the sugar transporters *EDR6 (Cla97C05G087120)* and *STP (Cla97C01G018840)* showed decreasing trends at early stages, but during the last stages of fruit development, a significant increase in their expression was observed, suggesting their roles in increasing sugar transport and accumulation (Fig. 4).

### Genes linked to the metabolism of organic acids

Malic acid is the key acid in watermelon and is thought to play a critical role in developing the sour taste and reducing the pH^12^. In total, 8 *ALMT* genes were found in the transcriptome data, but only *ALMT7 (Cla97C07G128420)* was consistent with the brown module and had a significant association with malate content. The expression of this gene was higher in SrW than in SwtW. One gene, namely, ‘malate synthase’ *(Cla97C03G054690)*, involved in the synthesis of malate was more highly expressed in SrW than in SwtW. The transcript levels of 2 malate dehydrogenase genes *(Cla97C06G125760* and *Cla97C05G103110)* and 1 *PEPC* gene *(Cla97C11G223580)* increased at early developmental stages but remained stable later. One gene for transmembrane malate transport *(Cla97C02G049340)* was also found to be upregulated. For malate degradation, 2 pyruvate kinase genes *(Cla97C04G076530* and *Cla97C11G218660)* showed a considerable increase in expression. One citrate synthase gene (*Cla97C03G068240*) was found in the brown module, and 1 isocitrate dehydrogenase gene (*Cla97C01G008870*) was found in the transcriptome data related to citric acid synthesis. The expression of these 2 genes was consistent with the citric acid content in SrW and SwtW at 4 developmental stages (Fig. 4).

### Validation of candidate genes in different watermelon accessions

**Fig. 5:**
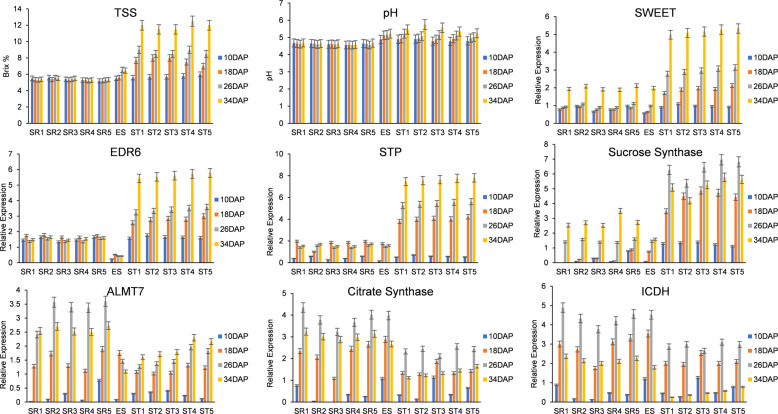
Measurement of pH, TSS, and qRT-PCR expression of candidate genes linked to sugars and organic acids in eleven watermelon accessions at different developmental stages of watermelon fruit

For further validation of candidate genes linked to sugar and organic acid transport, the expression profiles were investigated in 11 watermelon accessions. These 11 genotypes were selected based on the genetic diversity of sugars and organic acids to investigate the patterns of expression in diverse types of watermelon. Among these genotypes, Egusi and Amarus have low TSS and pH values, the edible-seed watermelon has low TSS and high pH values, and the improved lines have high TSS and pH values. The expression profiles of 7 key candidates (genes) identified from coexpression networks were checked via RT-qPCR at 4 developmental stages, i.e., 10, 18, 26, and 34 DAP. The genes linked to acids showed higher expression in accessions with low TSS and pH values. The genes linked to sugar transport were highly expressed in accessions with high pH and TSS values, which is consistent with the low acid contents and high sugar content. Thus, our results confirm that SWEET, STP, ERD6, SS, ALMT7, CS, and ICDH are potential candidate genes linked to sugar and organic acid transport and regulation during watermelon fruit development (Fig. 5).

## Discussion

The development and maturation of fruit are composite biological processes that are regulated by various environmental, hormonal, and gene regulation factors^7^. Sugar and acid regulatory pathways are vital metabolic constituents during fruit development and maturation. In this study, by using WGCNA for the first time in watermelon and integrating transcriptome and metabolome data, we identified complex genetic factors underlying variations in sugar and organic acid pathways in watermelon.

### Genetic factors underlying metabolic variations at various developmental stages

Our data showed that the fructose and sucrose contents were incredibly low in SwtW and SrW at the initial fruit development stages. Toward maturity, both of these compounds accumulated in large amounts, and a significant difference appeared between SwtW and SrW, especially for the sucrose content, which showed a substantial increase near maturity. Altogether, the fructose content was higher than the sucrose and glucose contents, which is similar to the findings of some earlier studies^12,34^. Likewise, our data showed that sugar metabolism in fruits mainly depends on developmental processes. The enzymes that regulate these extreme changes in sugar contents at different developmental stages, i.e., sucrose phosphate synthase (SPS), neutral invertase, fructose bisphosphate aldolase, sucrose synthase (SS), fructokinase (FK), and hexokinase (HK), exhibited varied expression at various developmental stages, which is consistent with the findings of the previous studies^35^. This consistency in developmental stage-dependent variation in genetic factors is also caused by variations in the sugar content itself. Sugar molecules, similar to hormones, act as signal regulators to alter gene expression levels. These signals are induced by the interaction of sugar molecules and sensor proteins^36^.

Organic acids usually exhibit high accumulation during the early developmental stages of fruit and tend to decrease near maturity; a similar trend was observed in this study. This decrease in organic acids at later stages results from the enhancement of sugar synthesis and secondary metabolic pathways towards ripening^37^. In this study, early accumulations of malate and citrate were observed, and as the maturity process continued, the malate and citrate contents decreased; these acids showed similar trends, but their points of degradation occurred early during development. These results were consistent with those of studies conducted on different fruits, including apple, peach, plum, and loquat^38,39^. Physiological changes during watermelon fruit development lead to substantial changes in fruit size, weight, flesh firmness, TSS, SSC, and acid content, thus affecting the overall quality of watermelon fruit. Therefore, it is vital to identify the key genetic factors, including gene networks and major contributors, controlling variations in these compounds, especially sugars and organic acids.

### Gene networks regulating sugar and organic acid metabolism

Complex biochemical pathways such as sugar synthesis and organic acid synthesis are regulated by multigene responses and cannot be explained by individual genes. Genes in these metabolic pathways are often regulated via coordinated expression, and hence, correlation-based models are often applied to identify gene networks. Previous studies have characterized several genes regulating sugars and organic acids in watermelon and other fruits. Some examples are the tonoplast sugar transporter *ClTST2* in watermelon^40^, tonoplast sugar transporter *PpTST1* in peach^41^, and tonoplast sugar transporter *CsPH8* and citrus transcription factor *CitVHA-c4* in citrus fruits^42^. Similarly, *ALMT-*family genes have often been reported to be involved in organic acid regulation in fruits and other plant parts^43–45^. A notable finding in all these reports is that these genes only slightly contribute to the overall variation in sugar and/or organic acid content because these complex metabolic traits are regulated by gene networks. We identified 11 gene modules/networks with WGCNA (a weighted-gene correlation module), among which 3 were highly correlated with sugars and organic acids.

Candidate genes for sugar metabolism in watermelon during the fruit maturation process mainly determine sweetness at the harvesting stage. Sugar molecules have to move across membranes during their transport into the chloroplast^46^, the vacuoles^47^, and the Golgi apparatus^48^. Multiple transporter genes are involved in this movement of sugars across membranes; for example, *SWEET* proteins form a distinct transporter family that consists of 17 members in *Arabidopsis* and 21 in rice. These members can transport sugars and are involved in the loading and storage of sugar molecules^49,50^. Similarly, proteins from the *MST* subfamily *ERD6* or *ESL1* are possibly involved in energy-independent sugar efflux from the vacuole^51,52^. In watermelon, however, there is little information available for the gene networks regulating sugar transport, the candidate genes linked to these networks, and their transcriptional regulation. We identified gene networks based on the coexpression of genes at the transcriptional level and their correlations with sugar content variation at various developmental stages. Within these networks, we found 3 genes (*Cla97C01G000640, Cla97C05G087120*, and *Cla97C01G018840*) annotated as sugar transporters, namely, *SWEET*, *EDR6*, and *STP*, and 1 gene (*Cla97C03G064990*) for sucrose synthesis. These genes were identified as hub genes within the modules, and the expression profiles in diverse watermelon accessions were consistent with the sugar content at various developmental stages. Thus, our confidence in these genes being the true variants for sugar transport is extremely high. None of these genes have been reported or studied in previous watermelon research and, therefore, should be investigated thoroughly in future molecular biology studies to fully understand the genetic regulators of sugar metabolism in watermelon.

Phosphoenolpyruvate carboxylase, aconitase (*ACO*), citrate synthase (*CS*), malate dehydrogenase (*MDH*), isocitrate dehydrogenase (*ICDH*), and malic enzyme (*ME*) are enzymes linked to organic acid metabolism and play a vital role in the biosynthesis and degradation of organic acids in fruit. Malic acid, a major organic acid in watermelon, is manufactured during the TCA cycle during different fruit growth and developmental stages. This acid is degraded during gluconeogenesis, and the TCA cycle takes place in the cytosol and mitochondrion. The vacuole is the major storage organ for malate.

We found that the brown module from WGCNA correlated significantly (*r*^2^ *=* 0.75) with the malate content. From the network built from the top genes in the brown module, *Cla97C07G128420* was identified as a hub gene, which encodes an *ALMT-*family protein. This gene was also identified as a differentially expressed gene between sweet and sour materials, and its relative quantitative expression also showed it to be consistently regulated with the malate content at various developmental stages. *ALMT* genes have also been reported in other crops to regulate organic acid contents, for example, *AtALMT9* and *AtALMT6* in *Arabidopsis thaliana* and four *AtALMT9* homologs in grape berries^53^. Similarly, in apple, genes linked to fruit acidity and/or pH include two *ALMT*-like genes, and *ALMT II* has already been reported^54,55^. According to these literature data and our findings, it is highly likely that *Cla97C07G128420* is a key candidate in the malate pathway’s gene network that contributes to the maximum trait variation. Similarly, *Cla97C01G008870* annotated as *ICDH* and *Cla97C03G068240* as citrate synthase from the brown module exhibited relative expression complementary to the citric acid content at all 4 developmental stages under investigation (Supplementary Fig. 4). These results suggest that these 3 genes are key players in the TCA cycle and pH regulation in watermelon. Fully characterizing these genes using molecular biology techniques would deepen our understanding of organic acid metabolism in watermelon.

### Potential genes in sugar and organic acid pathways not detected as key candidates

As mentioned earlier, we predicted key candidate genes according to a) hub genes from modules correlated significantly with fructose, glucose, malic acid, and citric acid and b) the expression profiles of selected genes to narrow down the search. However, several other genes encoding proteins for sugar and organic acid pathways also showed differential expression between diverse watermelon types. For example, *Cla97C05G094630*, which is associated with fructose kinase protein, showed downregulated expression, which would suggest that fructose is less utilized during the metabolic process^56^. Likewise, higher accumulation of fructose was observed at the maturity stage in our material, which suggests that *Cla97C05G094630* does play a vital role in fructose regulation; however, this gene was not identified as a potential candidate based on intramodular gene significance. Similarly, we found two hexokinase-related genes (*Cla97C03G063270* and *Cla97C11G208480*) showing upregulated expression at maturity. Hexokinases are commonly known to be involved in the glycolysis process; however, these genes were not among the high-confidence genes based on our selected models. The accumulation of sucrose is primarily linked with the activity of sucrose-metabolizing enzymes, including *SPS*, and *sucrose synthase*^57^. Two *SPS*-related genes (*Cla97C11G215270* and *Cla97C06G120770*) and one sucrose synthase gene (*Cla97C03G064990*) were found in the blue module, which was positively correlated with sugar content. The relative expression of these genes also complemented the sugar content at various developmental stages, but their intramodular significance was not exceedingly high. *Cla97C05G103110*, identified in the brown module and correlated with malate content, is associated with the NAD-MDH enzyme based on available data from other crops. This gene also showed expression consistent with the malate content during various developmental stages in our study. Other important genes, such as *Cla97C03G066480* (malic enzyme), *Cla97C11G223580* (*PEPCK*), *Cla97C04G076530*, and *Cla97C11G218660* (both annotated for pyruvate kinases), also showed expression trends consistent with changes in relevant traits.

These results indicate that complex metabolic pathways such as those for sugars and organic acids regulate the coordinated expression of gene networks and that certain genes within the networks make key contributions to trait variation. One limitation of our study should also be considered: the statistical power of our analysis was low due to the small sample size. Nevertheless, the expression profiling of key candidates in genetically diverse accessions supported our findings. In future studies, larger and more diverse samples should be investigated to further demonstrate the coexpression patterns of genes/gene networks. Additionally, future studies should focus on how certain changes in the gene networks can affect other pathways or traits, and to do so, high-throughput phenotyping of agronomic and metabolic traits should be incorporated into the analysis.

## Conclusions

In the present study, we used transcriptome profiles to investigate gene networks (based on coexpression patterns) controlling the regulation of sugars and organic acids in watermelon. We identified 3 coexpression modules/gene networks that were highly correlated with variations in the sugar and organic acid contents at various developmental stages. Within these networks, we identified 7 key candidate genes that were weighted as module hub genes, and their quantitative expression was correlated with phenotypic variation. The expression profiles of these 7 genes in genetically diverse watermelon accessions further supported our findings. In total, we identified 4 candidate genes for sugar content and 3 for organic acid content. The multiple and stringent screening steps used in our study increased the probability of and confidence in these genes being true candidates in the networks controlling sugar and organic acid pathways. These genes are newly identified, and no previous reports about their role or functions in watermelon are available.

### Core ideas

- The integration of metabolic phenotypes and gene expression profiles can reveal gene networks and key candidate genes regulating metabolic variations.
- This study reports 4 new high-confidence candidate genes (*Cla97C01G000640*, *Cla97C05G087120*, *Cla97C01G018840* and *Cla97C03G064990*) controlling sugar transport and regulation in watermelon.
- This study also reports 3 new high-confidence candidate genes (*Cla97C07G128420*, *Cla97C03G068240*, and *Cla97C01G008870*) for malic acid and citric acid regulation in watermelon.

## Supplementary information

Supplementary Figure 1

Supplementary Figure 2

Supplementary Figure 3

Supplementary Figure 4

Supplementary Figure 5

Supplementary Table 1

Supplementary File 1

## Data Availability

The authors declare that the data supporting the study findings are presented in the article and Supplementary Information files or are available from the corresponding author upon request.

## References

[1] Faostat, F. Available online: http://www.fao.org/faostat/en/#data. *QC (accessed on January 2018)* (2017).

[2] Guo, S. et al. Resequencing of 414 cultivated and wild watermelon accessions identifies selection for fruit quality traits. *Nat. Genet.* **51**, 1–8 (2019). 10.1038/s41588-019-0518-4 31676863

[3] Akashi K (2017). Spatial accumulation pattern of citrulline and other nutrients in immature and mature watermelon fruits. J. Sci. Food Agric..

[4] Hayashi T (2005). l-Citrulline and l-arginine supplementation retards the progression of high-cholesterol-diet-induced atherosclerosis in rabbits. Proc. Natl Acad. Sci. USA.

[5] Collins JK (2007). Watermelon consumption increases plasma arginine concentrations in adults. Nutrition.

[6] Saminathan T (2018). Metagenomic and metatranscriptomic analyses of diverse watermelon cultivars reveal the role of fruit associated microbiome in carbohydrate metabolism and ripening of mature fruits. Front. Plant Sci..

[7] Wechter WP (2008). Gene expression in developing watermelon fruit. BMC Genomics.

[8] Borsani J (2009). Carbon metabolism of peach fruit after harvest: changes in enzymes involved in organic acid and sugar level modifications. J. Exp. Bot..

[9] Zhu Q (2017). Comparative transcriptome analysis of two contrasting watermelon genotypes during fruit development and ripening. BMC Genomics.

[10] Montero TM, Mollá EM, Esteban RM, López-Andréu FJ (1996). Quality attributes of strawberry during ripening. Sci. Horticulturae.

[11] Jiang CC, Fang ZZ, Zhou DR, Pan SL, Ye XF (2019). Changes in secondary metabolites, organic acids and soluble sugars during the development of plum fruit cv.‘Furongli’(Prunus salicina Lindl). J. Sci. Food Agric..

[12] Jawad UM (2020). Expression pattern of sugars and organic acids regulatory genes during watermelon fruit development. Sci. Horticulturae.

[13] Miron D, Schaffer AA (1991). Sucrose phosphate synthase, sucrose synthase, and invertase activities in developing fruit of Lycopersicon esculentum Mill. and the sucrose accumulating Lycopersicon hirsutum Humb. and Bonpl. Plant Physiol..

[14] Granot D, David-Schwartz R, Kelly G (2013). Hexose kinases and their role in sugar-sensing and plant development. Front. Plant Sci..

[15] D'Ambrosio C (2013). Proteomic analysis of apricot fruit during ripening. J. Proteom..

[16] dos Santos RS (2015). Genetic regulation and the impact of omics in fruit ripening. Plant Omics.

[17] Langfelder P, Horvath S (2008). WGCNA: an R package for weighted correlation network analysis. BMC Bioinf..

[18] Bai Y, Dougherty L, Cheng L, Zhong G-Y, Xu K (2015). Uncovering co-expression gene network modules regulating fruit acidity in diverse apples. BMC genomics.

[19] Zhang Q (2019). Transcriptional regulatory networks controlling taste and aroma quality of apricot (Prunus armeniaca L.) fruit during ripening. BMC genomics.

[20] Li H (2020). MdMYB8 is associated with flavonol biosynthesis via the activation of the MdFLS promoter in the fruits of Malus crabapple. Horticulture Res..

[21] Gao L (2018). Comparative transcriptome analysis reveals key genes potentially related to soluble sugar and organic acid accumulation in watermelon. PLoS ONE.

[22] Bartolozzi F, Bertazza G, Bassi D, Cristoferi G (1997). Simultaneous determination of soluble sugars and organic acids as their trimethylsilyl derivatives in apricot fruits by gas-liquid chromatography. J. Chromatogr. A.

[23] Shi C-Y (2015). Citrus PH5-like H+-ATPase genes: identification and transcript analysis to investigate their possible relationship with citrate accumulation in fruits. Front. Plant Sci..

[24] Wang N (2018). Transcriptomic analysis of red-fleshed apples reveals the novel role of MdWRKY11 in flavonoid and anthocyanin biosynthesis. J. Agric. Food Chem..

[25] Anders S, Pyl PT, Huber W (2015). HTSeq—a Python framework to work with high-throughput sequencing data. Bioinformatics.

[26] Mortazavi A, Williams BA, McCue K, Schaeffer L, Wold B (2008). Mapping and quantifying mammalian transcriptomes by RNA-Seq. Nat. Methods.

[27] Andino GK, Gribskov M, Anderson DL, Evans JD, Hunt GJ (2016). Differential gene expression in Varroa jacobsoni mites following a host shift to European honey bees (Apis mellifera). BMC Genomics.

[28] Benjamini Y, Hochberg Y (1995). Controlling the false discovery rate: a practical and powerful approach to multiple testing. J. R. Stat. Soc.: Ser. B (Methodol.).

[29] Anders, S. & Huber, W. Differential expression analysis for sequence count data. *Nature Preceedings* **11**, R106 (2010). 10.1186/gb-2010-11-10-r106 PMC3218662 20979621

[30] Mao X, Cai T, Olyarchuk JG, Wei L (2005). Automated genome annotation and pathway identification using the KEGG Orthology (KO) as a controlled vocabulary. Bioinformatics.

[31] Zhang, B. & Horvath, S. A general framework for weighted gene co-expression network analysis. *Stat. Appl. Genet. Mol. Biol.* **4**, 17(2005). 10.2202/1544-6115.1128 16646834

[32] Kariyanna B (2020). Identification of suitable reference genes for normalization of RT-qPCR data in eggplant fruit and shoot borer (Leucinodes orbonalis Guenée). Biologia.

[33] Kong, Q. et al. Identification of suitable reference genes for gene expression normalization in qRT-PCR analysis in watermelon. *PloS ONE* **9**, e90612 (2014). 10.1371/journal.pone.0090612 PMC3938773 24587403

[34] Gao, L. et al. Comparative transcriptome analysis reveals key genes potentially related to soluble sugar and organic acid accumulation in watermelon. *PLoS ONE* **13**, e0190096 (2018). 10.1371/journal.pone.0190096 PMC5764247 29324867

[35] Li, M., Feng, F. & Cheng, L. Expression patterns of genes involved in sugar metabolism and accumulation during apple fruit development. *PLoS ONE* **7**, e33055 (2012). 10.1371/journal.pone.0033055 PMC3296772 22412983

[36] Smeekens S (2000). Sugar-induced signal transduction in plants. Annu. Rev. Plant Biol..

[37] Lombardo VA (2011). Metabolic profiling during peach fruit development and ripening reveals the metabolic networks that underpin each developmental stage. Plant Physiol..

[38] Famiani F, Battistelli A, Moscatello S, Cruz-Castillo JG, Walker RP (2015). The organic acids that are accumulated in the flesh of fruits: occurrence, metabolism and factors affecting their contents-a review. Rev. Chapingo Ser. Horticultura.

[39] Yao Y-X (2009). Molecular cloning of three malic acid related genes MdPEPC, MdVHA-A, MdcyME and their expression analysis in apple fruits. Sci. Horticulturae.

[40] Ren Y (2018). A tonoplast sugar transporter underlies a sugar accumulation QTL in watermelon. Plant Physiol..

[41] Peng Q (2020). Functional analysis reveals the regulatory role of PpTST1 encoding tonoplast sugar transporter in sugar accumulation of peach fruit. Int. J. Mol. Sci..

[42] Li S-j (2016). The Citrus transcription factor, CitERF13, regulates citric acid accumulation via a protein-protein interaction with the vacuolar proton pump, CitVHA-c4. Sci. Rep..

[43] De Angeli A (2013). The vacuolar channel VvALMT9 mediates malate and tartrate accumulation in berries of Vitis vinifera. Planta.

[44] Etienne A, Génard M, Lobit P, Mbeguié-A-Mbéguié D, Bugaud C (2013). What controls fleshy fruit acidity? A review of malate and citrate accumulation in fruit cells. J. Exp. Bot..

[45] Etienne C (2002). Candidate genes and QTLs for sugar and organic acid content in peach [Prunus persica (L.) Batsch]. Theor. Appl. Genet..

[46] Weber A (2000). Identification, purification, and molecular cloning of a putative plastidic glucose translocator. Plant Cell.

[47] Martinoia E, Massonneau A, Frangne N (2000). Transport processes of solutes across the vacuolar membrane of higher plants. Plant Cell Physiol..

[48] Wang HX (2006). A Golgi-localized hexose transporter is involved in heterotrimeric G protein-mediated early development in Arabidopsis. Mol. Biol. Cell.

[49] Chen L-Q (2012). Sucrose efflux mediated by SWEET proteins as a key step for phloem transport. Science.

[50] Chardon F (2013). Leaf fructose content is controlled by the vacuolar transporter SWEET17 in Arabidopsis. Curr. Biol..

[51] Klemens PA (2014). Overexpression of a proton‐coupled vacuolar glucose exporter impairs freezing tolerance and seed germination. N. phytologist.

[52] Poschet G (2011). A novel Arabidopsis vacuolar glucose exporter is involved in cellular sugar homeostasis and affects the composition of seed storage compounds. Plant Physiol..

[53] Terrier N, Deguilloux C, Sauvage F-X, Martinoia E, Romieu C (1998). Proton pumps and anion transport in Vitis vinifera: the inorganic pyrophosphatase plays a predominant role in the energization of the tonoplast. Plant Physiol. Biochem..

[54] Ma, B. et al. Genes encoding aluminum-activated malate transporter II and their association with fruit acidity in apple. *The Plant Genome* **8**, 1–14 (2015). 10.3835/plantgenome2015.03.0016 33228269

[55] Bai Y (2012). A natural mutation-led truncation in one of the two aluminum-activated malate transporter-like genes at the Ma locus is associated with low fruit acidity in apple. Mol. Genet. genomics.

[56] Berüter J, Feusi MES, Rüedi P (1997). Sorbitol and sucrose partitioning in the growing apple fruit. J. Plant Physiol..

[57] Ludewig F, Flügge U-I (2013). Role of metabolite transporters in source-sink carbon allocation. Front. Plant Sci..

