# Supplementary figures and images for: Identification of key gene networks controlling organic acid and sugar metabolism during watermelon fruit development by integrating metabolic phenotypes and gene expression profiles
Source: Hortic Res. 2020 Dec 1;7:193. doi: 10.1038/s41438-020-00416-8 (PMC7705761; doi:10.1038/s41438-020-00416-8)

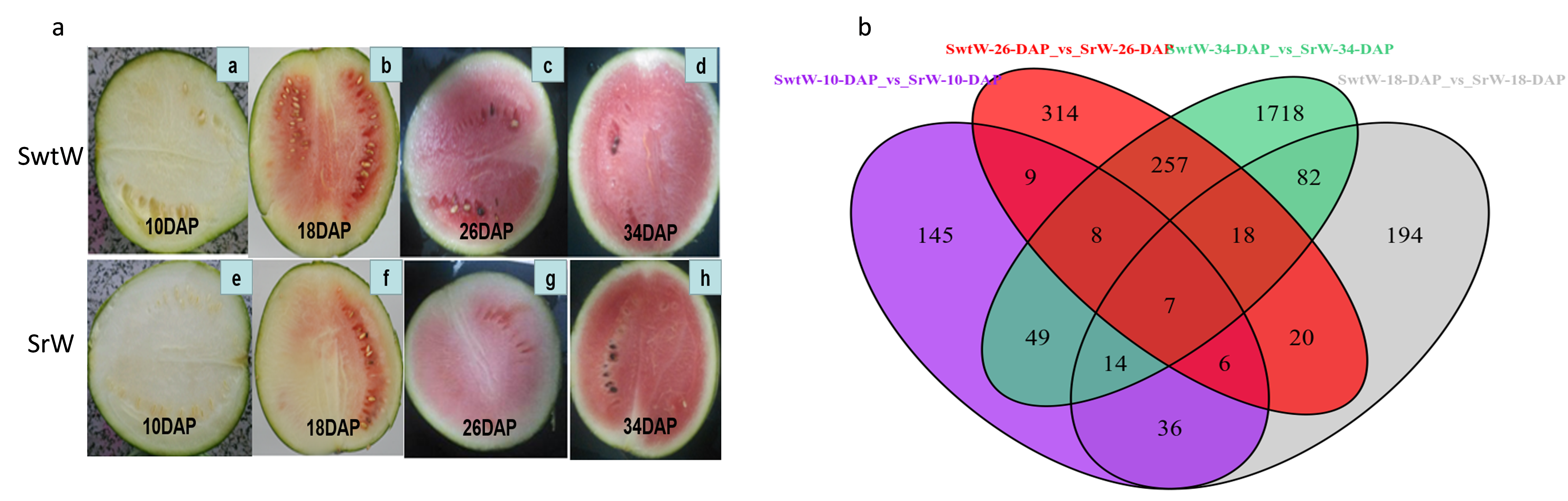

Supplement: Supplementary file 1 — Supplementary Figure 1 [file 41438_2020_416_MOESM1_ESM.png]

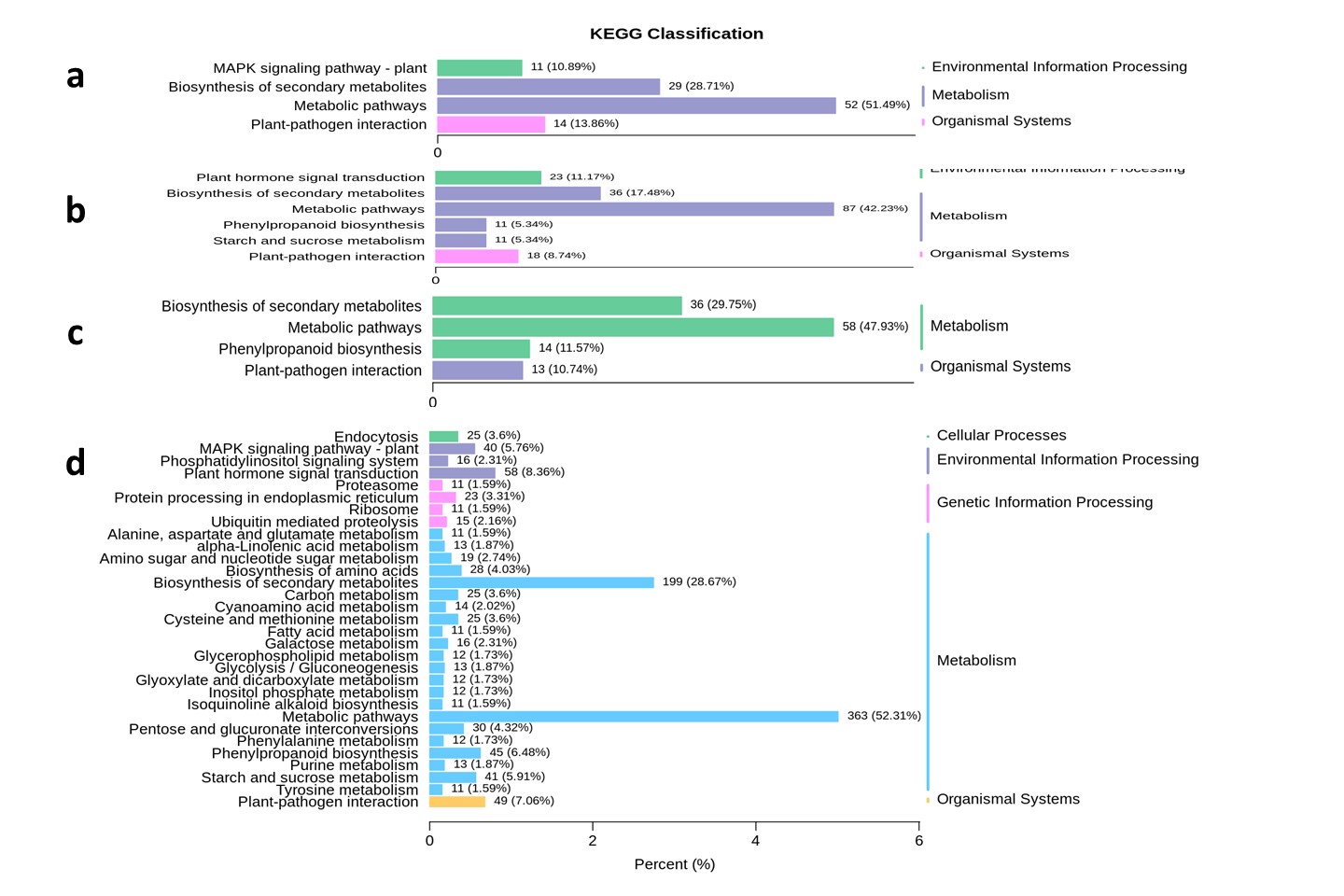

Supplement: Supplementary file 2 — Supplementary Figure 2 [file 41438_2020_416_MOESM2_ESM.jpg]

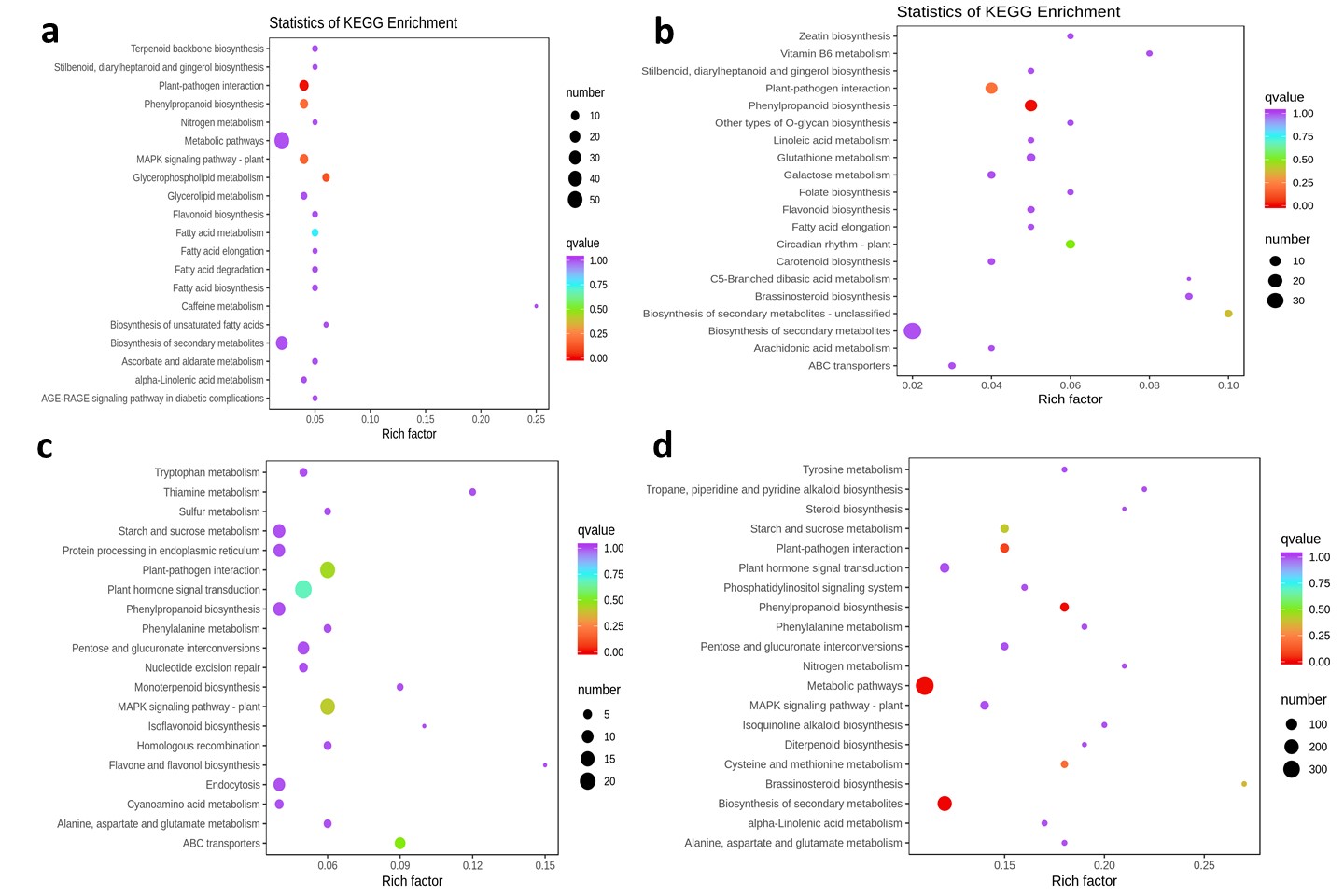

Supplement: Supplementary file 3 — Supplementary Figure 3 [file 41438_2020_416_MOESM3_ESM.jpg]

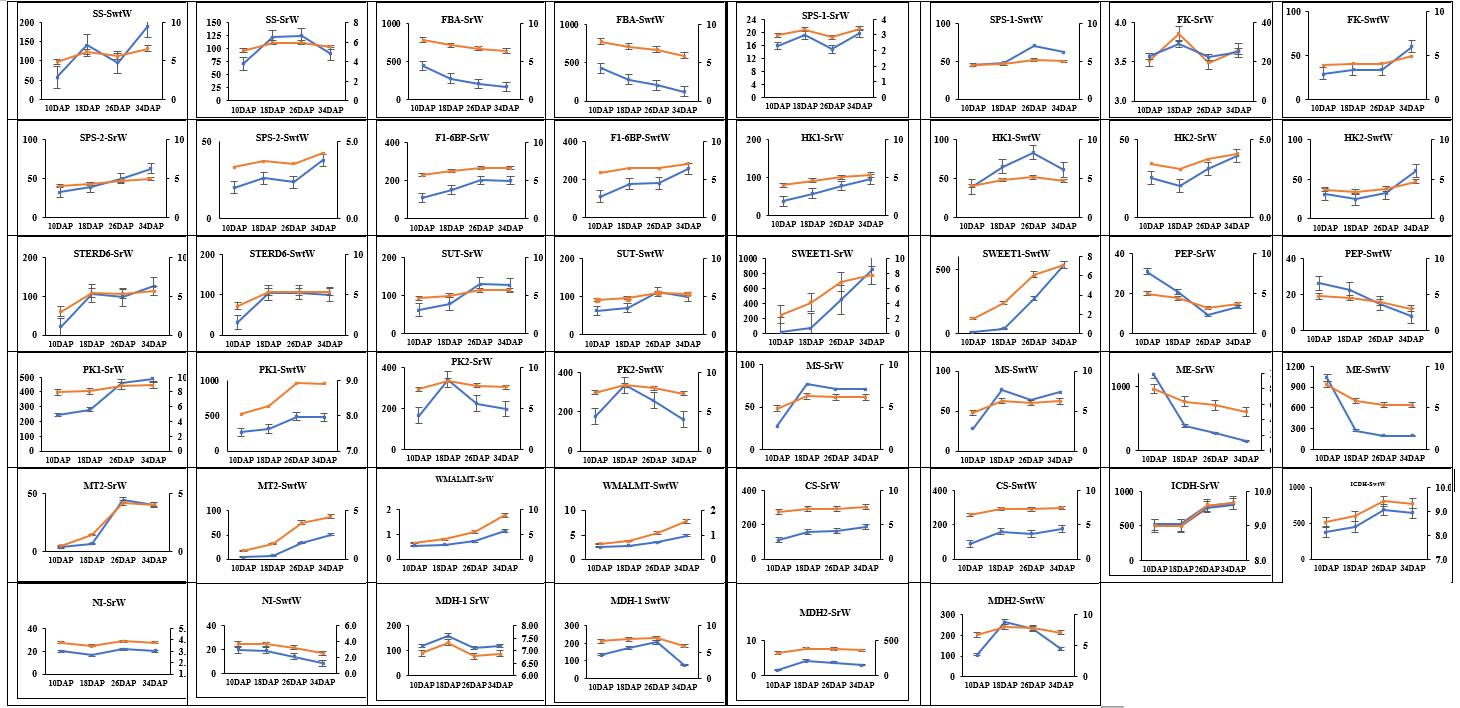

Supplement: Supplementary file 4 — Supplementary Figure 4 [file 41438_2020_416_MOESM4_ESM.jpg]

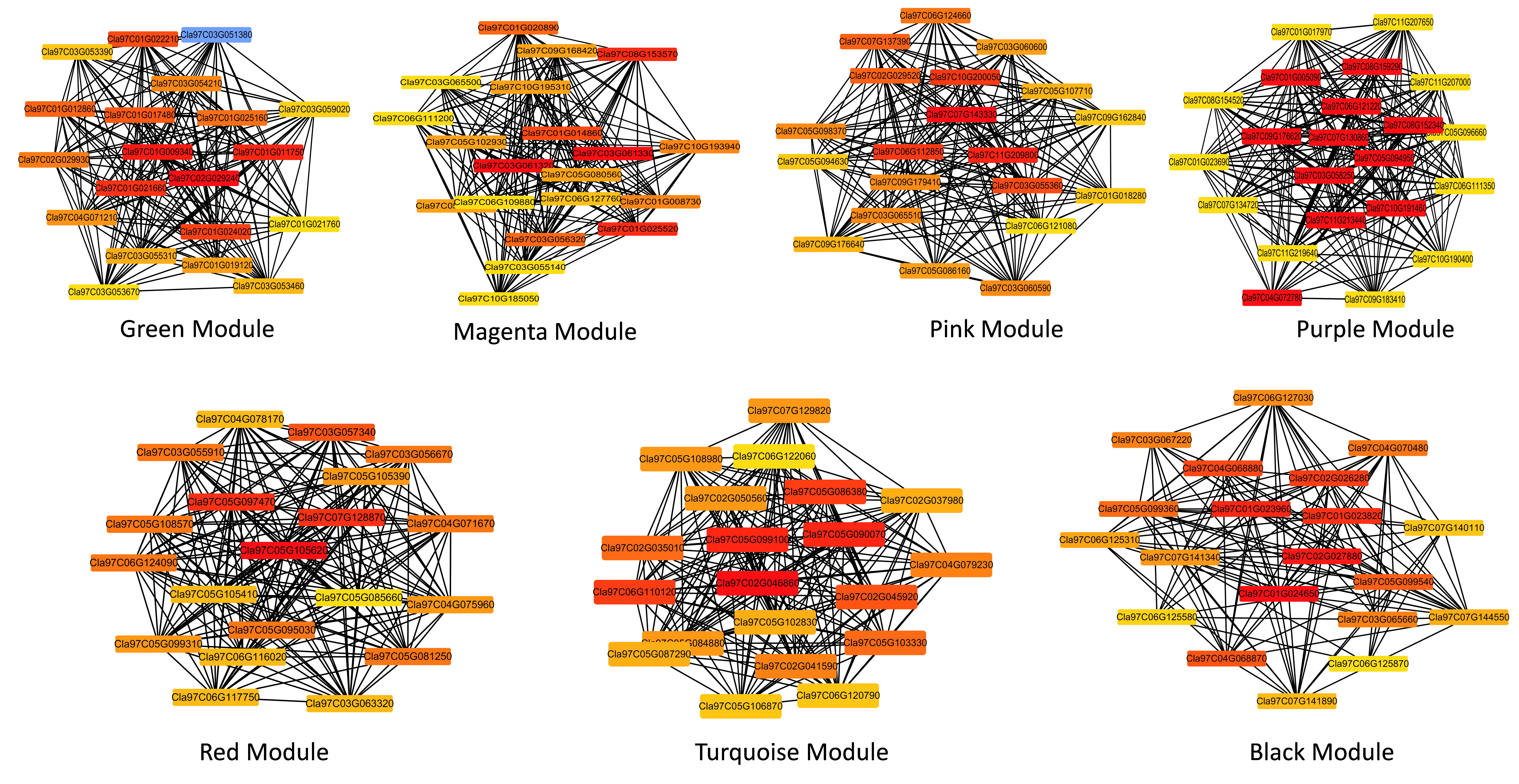

Supplement: Supplementary file 5 — Supplementary Figure 5 [file 41438_2020_416_MOESM5_ESM.png]
