# Supplementary material for: Identification of key gene networks controlling organic acid and sugar metabolism during watermelon fruit development by integrating metabolic phenotypes and gene expression profiles
Source: Hortic Res. 2020 Dec 1;7:193. doi: 10.1038/s41438-020-00416-8 (PMC7705761; doi:10.1038/s41438-020-00416-8)
Supplement: Supplementary file 6 — Supplementary Table 1 [file 41438_2020_416_MOESM6_ESM.docx]

**Supplementary Table 1:** Primers used for q-RT-PCR

| **Gene ID** | **Forward Primer** | **Reverse Primer** |
| --- | --- | --- |
| **Cla97C03G064990-SS** | GGAAAAGGAATCCTGCAACA | AACCAGTGCATGGACATTGA |
| **Cla97C06G120770-SPS2** | GGAGAAAGTGGGGACACAGA | GGCACTGGTAGCCTCTTCAG |
| **Cla97C11G215270-SPS1** | GAATTTGATGCGTTGGTGTG | CGTTCCCACCTTCCAACTTA |
| **Cla97C04G076620-FBA** | GCCAGGAGAATGGTCTGGTA | TGCAACCTTTGCAGATTGAG |
| **Cla97C05G094630-FK** | GCAAACGATTTCACCACCTT | CACAAGCAAGAAACCCATGA |
| **Cla97C08G147060-F1-6BP** | TTGTATGGCAGCTCTTGCAC | ACTTTGCAGTTGGACCATCC |
| **Cla97C05G088310-NI** | TCTACAGCCTGCTCACATGG | GCCAGTTATTATGCGCCATT |
| **Cla97C03G063270-HK1** | GGTGGGGGTTTTAGAAGAGC | CCCCAAGATCCAGAGCATAA |
| **Cla97C11G208480-HK2** | GCCTCTCTGCTGCTGGTATC | TTGAATGCTCGATGAAGACG |
| **Cla97C01G000640-SWEET1** | TTGGCCTCGTTGCTTCTATT | TGGAGACGCAAGGAAGAGAT |
| **Cla97C04G076530-PK1** | GCATTTGAGGCTAGGCAGTC | TCCCCAACCTTCTGACAAAC |
| **Cla97C11G218660-PK2** | AAGATCATGGCTCGGATTTG | AACCAACTTGGCTGTGGTTC |
| **Cla97C06G125760-MDH1** | CCTCAAGGCAGCTTCATCTC | GAGAAGTGCCAAAGGCTGAC |
| **Cla97C05G103110-MDH2** | CTCTGAAAGCGGGTGTCTTC | TTCCCCATTTTTCAATCAGC |
| **Cla97C03G066480-ME** | TGCGCTTAAACTCATTGGTG | CAGATCTTCTTGCGGGTCTC |
| **Cla97C02G049340-MT2** | GACCGGACTTGGTAATCGAA | CATCACCAACATTGCTACCG |
| **Cla97C03G054690-MS** | TTTGTGGCTGATTTGCAGAG | ATGATCATCTTCCGCTCCAC |
| **Cla97C07G128420-ALMT** | TCTGATGCCAGTTTCAGCAC | TCTGGGGGATTGAAGACAAG |
| **Cla97C11G223580-PEP** | AACCACCGGAGACAGTTCAC | GTGAAACTGGAGGGTGCATT |
| **Cla97C01G008870-ICDH** | GGGCGTGCAAGAACTATGAT | ACGAGACCAAGCAAAAATGG |
| **Cla97C03G068240-CS** | TTTGGGAAACGTGGAAAGAG | AGCCAAGCCTTTCAGAAACA |
| **Cla97C05G087120-STERD6** | ATTACCCTTCTTGCGGGTCT | TGATTTTGGGGAGACTTTGC |
| **Cla97C01G018840-SUT** | CGCTACAAGTTTTGGGCATT | TCCATCTCAAGCACATCGAG |
